# Environmental Risk Assessment of Polycyclic Aromatic Hydrocarbons in Farmland Soils near Highways: A Case Study of Guangzhou, China

**DOI:** 10.3390/ijerph191610265

**Published:** 2022-08-18

**Authors:** Xiaorong Zhang, Weiqing Lu, Linyu Xu, Wenhao Wu, Bowen Sun, Wenfeng Fan, Hanzhong Zheng, Jingjing Huang

**Affiliations:** State Key Joint Laboratory of Environmental Simulation and Pollution Control, School of Environment, Beijing Normal University, No. 19, Xinjiekouwai Street, Haidian District, Beijing 100875, China

**Keywords:** polycyclic aromatic hydrocarbons (PAHs), traffic sources, farmland soil, seasonal variations, risk

## Abstract

Recently, the rapid growth in vehicle activity in rapidly urbanized areas has led to the discharge of large amounts of polycyclic aromatic hydrocarbons (PAHs) into roadside soils and these compounds have gradually accumulated in the soil, which poses a serious threat to national food security and public health. However, previous studies did not clearly investigate the seasonal differences in PAH pollution of roadside soil by different highways. Therefore, based on field investigations, this study collected 84 soil surface samples to compare the pollution characteristics of 16 PAHs in farmland soils located near different roads in different seasons in Guangzhou, China. The results showed that the concentration of Σ16PAHs in farmland soils in spring (with a mean value of 258.604 μg/kg) was much higher than that in autumn (with a mean value of 157.531 μg/kg). There are differences in the PAH compositions in spring (4 ring > 3 ring > 5 ring > 6 ring) and autumn (4 ring > 5 ring > 6 ring > 3 ring). The proportion of 4–6 ring PAHs was much higher than 2–3 ring PAHs in both seasons. The spatial differences were significant. The sampling areas with higher concentrations of 16 PAHs were Tanbu Town, Huadu District (TB), Shitan Town, Zengcheng District (ST), and Huashan Town, Huadu District (HS), while the lowest concentration was in Lanhe Town, Nansha District (LH). The results of the diagnostic ratios showed that the main source of soil PAHs consists of a mixed source from petroleum and biomass combustion. The results from the total pollution assessment method and Nemerow index method indicated that the pollution levels of PAHs in the farmland soils indicated weak contamination. Our study provides a scientific basis for the prevention and control of soil pollution in farmlands near highways.

## 1. Introduction

With the development of the global economy and acceleration of urbanization, urban transportation facilities have continuously improved, and the number of vehicles has increased rapidly. Vehicle exhaust emissions, as a typical anthropogenic source of polycyclic aromatic hydrocarbons (PAHs), accumulate in soil through wet and dry deposition, surface runoff, and other ways, which cause serious pollution of surface soils and affect food safety and human health by crop absorption and incorporation into the food chain [1,2,3]. In addition, the continuous expansion of transportation facilities has led to unbalanced land use, much traffic land has occupied cultivated land, and large amounts of farmland are still reserved along the highways. Soils play an important role in ensuring the sustainable development of a country. Especially in cultivated soils, the soil quality is closely related to agricultural product safety and human health. PAHs have carcinogenic, teratogenic, and mutagenic effects and can enter the human body through ingestion, dermal contact, and inhalation to cause various diseases [4]. These are some of the most harmful environmental pollutants with respect to human health, and 16 PAHs were listed as pollutants with priority control by the United States Environmental Protection Agency (U.S. EPA) [5,6,7,8,9]. Some studies have found that soils bear more than 90% of the environmental load of PAHs and are a potential source and sink in the environment [10,11,12,13,14]. Therefore, it is necessary to explore the pollution of farmland soil PAHs by traffic emissions and to assess their environmental risks.

Many scholars have paid attention to the influencing factors of soil PAH pollution and found that urbanization is an important driving factor [15,16]. Urbanization has led to the rapid centralization of large populations, transportation infrastructures, and an increase in the number of vehicles and industrial activities, which have severely affected the distribution characteristics of soil PAHs [17]. For example, the traffic emissions and coal and biomass combustion that occur due to the rapid urbanization and industrialization processes also lead to the high PAH concentrations that are found in the urban topsoils of China [18]. Some scholars have also conducted research on soil PAH pollution levels and pollution sources [19,20,21]. For example, some scholars studied PAHs in the soils of two cities in Florida and found that the PAH concentrations in the central districts and near roads with high traffic volumes were high [22]. Moreover, industrial sources and traffic sources were also found to be important sources of soil PAHs, and the PAH concentrations in the soils near traffic areas and industrial areas were five times those found in the suburbs [23]. In addition, some scholars have also analyzed the relationship between soil PAH pollution and traffic road. One study found that the PAH concentrations in soil were related to the distances to roads and to traffic congestion [2,24]. The study also found that the PAH concentrations gradually decreased with increasing distances from roads [25]. The soils near roadsides have higher PAH concentrations and have the most serious pollution [26].

In general, there are still deficiencies. First, although beneficial some studies have been conducted on the PAHs in soils along highways and major and minor roads, little attention has been given to the PAHs in farmland soils located near different highways. A systematic study on soil PAH pollution in different highway conditions is lacking. Second, few studies have focused on the seasonal drivers of PAH pollution characteristics and pollution levels in farmland soils along traffic roads. The high traffic densities in urban areas lead to serious PAH pollution in surface soils. Guangzhou, as China’s third largest city and core city of the Guangdong–Hong Kong–Macao Greater Bay Area, is one of the rapidly urbanized areas. The vehicle numbers in Guangzhou increased sharply from 0.035 million in 1999 to 3.08 million in 2020 (Guangzhou Statistical Yearbook, 2021). The fast-growing vehicle numbers and rapid increases in traffic congestion have exacerbated pollution due to PAHs in the farmland soils near highways. Accurate analysis of the pollution characteristics of soil PAHs and pollution levels is conducive to the formulation of risk mitigation strategies.

Therefore, it is necessary to assess the pollution characteristics and pollution levels of PAHs in farmland soils located near highways in different seasons. In our study, we collected 84 samples from farmland soil near different highways in different seasons. The specific purposes of this study were to (1) describe the relationship between traffic emissions and PAH pollution in farmland soils located near highways; (2) illustrate the seasonal differences in the concentrations and distributions of PAHs in farmland soils; and (3) identify the potential sources and assess the ecological and health risks of PAHs.

## 2. Materials and Methods

### 2.1. Study Area

Guangzhou is located in the Pearl River Delta in southeast China and is China’s third largest city, with an area of 7434 km^2^ and population of approximately 19 million. According to the Guangzhou Statistical Yearbook (2021), Guangzhou receives a large amount annual rainfall, with an annual average of 1800 mm. The rainfall amounts are highest in summer, which are followed by those in spring, autumn, and finally winter. Guangzhou is one of the central cities of the Guangdong–Hong Kong–Macao Greater Bay Area and has a complete transportation network. By 2020, 1102 km of expressways had been constructed, and car ownership had reached 3.08 million. With the development of rapid urbanization and traffic, Guangzhou is facing severe soil PAH pollution.

### 2.2. Soil Sampling and Analysis

**Figure 1:**
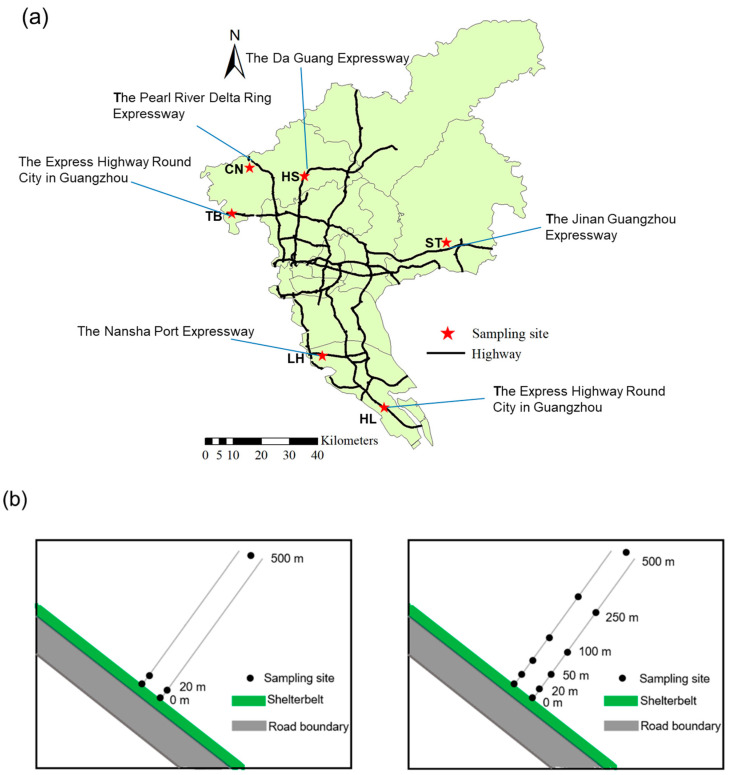
Sampling areas and sampling methods used in Guangzhou. (**a**) The sampling area; (**b**) the sampling method, where the left is non-gradient sampling (CN, HS, ST, LH) and the right is gradient sampling (TB, HL).

We try our best to avoid the impact of industrial emissions on soil PAHs. After the field investigation, we chose roads far away from the industrial area. In addition, factors such as Guangzhou’s traffic road network, agricultural planting areas, and vehicle driving conditions were comprehensively considered. Therefore, the farmland used for planting vegetables near the toll gates of 6 different highways was selected for the sampling site layout. In addition, when considering the temperatures in four seasons in Guangzhou, to avoid the influence of temperature on PAHs, we selected spring and autumn, which have similar average temperatures, for sample collection. The details of the 6 sampling areas are shown in Table 1. We adopted gradient sampling in the TB and HL areas and non-gradient sampling in the CN, HS, ST, and LH areas when collecting soil samples in autumn (during October 2020) and spring (during March 2021) (Figure 1). We collected 84 surface soil (0–20 cm) samples (10 samples in each area of CN, HS, ST, and LH, and 22 samples in each area of TB and HL) in autumn and spring, and 42 samples were collected in each season. In addition, each area is represented by a background value that is located 500 m away from the highways. We mixed five subsamples (e.g., 4 corner samples and 1 center sample) to form one 20 g composite sample for each site. The samples were stored in brown glass bottles before storage at 4 °C until further analysis by the testing company.

Before the chemical analyses, the soil samples were air dried at room temperature and were then sieved through a 100-mesh sieve to remove stones and plant residues. We adopted the U.S. EPA standard 3550C and U.S. EPA standard 3630C to extract and purify the soil PAHs [27,28,29]. The method was as follows. Firstly, 5 g of soil samples was mixed with anhydrous sodium sulfate, and then we used the Soxhlet extraction method to extract the target compound with a mixture of dichloromethane and n-hexane (*v*/*v* = 1:1). Secondly, we concentrated the extracts by rotary vacuum evaporation (at 35 °C). Thirdly, the solvent was changed to n-hexane, and the concentrated extracts were purified by adopting a glass column fitted with anhydrous sodium sulfate and silica gel. Next, a mixture of n-hexane/dichloromethane (*v*/*v* = 3:2) was used to elute the column. Finally, the collected PAH fraction was then concentrated to 1.0 mL under a gentle stream of nitrogen for measurement on a GC-MS instrument.

Gas chromatography–mass spectrometry (GC-MS) (6890N/5975B, Agilent, Santa Clara, CA, USA) was used to measure the concentrations of 16 PAHs. We used a fused silica capillary Rtx-5MS column (30 m × 0.25 mm inner diameter × 0.25 μm film thickness) to separate the different chemical compounds. The injection volume was 1 μL with splitless mode, and helium (purity > 99.999%) with a flow rate of 1 mL/min was the career gas. Initial oven temperature was programmed as 80 °C for 2 min, and increased to 180 °C at a rate of 20 °C/min for 5 min; then ramped to 290 °C at a rate of 10 °C/min, and maintained for 5 min. We chose electron impact (EI) mode to carry out ionization, and selective ion monitoring (SIM) mode to obtain data. We measured the 16 PAHs by the internal standard method. At the same time, strict quality control measures were employed in this process, and both blank and parallel samples were analyzed. The recovery rate of PAHs ranged between 70.2% and 110.8%.

### 2.3. Exposure Model

Human exposure to soil PAHs mainly occurs in three ways, namely, ingestion, dermal contact, and inhalation. The incremental lifetime cancer risk (ILCR) is usually used to assess the carcinogenic risks for children and adults who are exposed to PAHs in soils [27,28,30,31,32]. The ILCR formulas for the three pathways are shown below.

(1) $${\mathrm{ILCR}}_{\mathrm{ingestion}}=\left({\mathrm{CSF}}_{\mathrm{ingestion}}\times \sqrt[{3}]{\frac{\mathrm{BW}}{70}}\right)\times \frac{{\mathrm{CS}\times \mathrm{IR}}_{\mathrm{ingestion}}\times \mathrm{EF}\times \mathrm{ED}}{{\mathrm{BW}\times \mathrm{AT}\times 10}^{6}},$$

(2) $${\mathrm{ILCR}}_{\mathrm{dermal}}=\left({\mathrm{CSF}}_{\mathrm{dermal}}\times \sqrt[{3}]{\frac{\mathrm{BW}}{70}}\right)\times \frac{\mathrm{CS}\times \mathrm{SA}\times \mathrm{AF}\times \mathrm{ABS}\times \mathrm{EF}\times \mathrm{ED}}{{\mathrm{BW}\times \mathrm{AT}\times 10}^{6}},$$

(3) $${\mathrm{ILCR}}_{\mathrm{inhalation}}=\left({\mathrm{CSF}}_{\mathrm{inhalation}}\times \sqrt[{3}]{\frac{\mathrm{BW}}{70}}\right)\times \frac{{\mathrm{CS}\times \mathrm{IR}}_{\mathrm{inhalation}}\times \mathrm{EF}\times \mathrm{ED}}{\mathrm{BW}\times \mathrm{AT}\times \mathrm{PEF}},$$

where CS is the concentration of soil PAHs (μg/kg); CSF represents the carcinogenic slope factor (mg kg^−1^ day^−1^); and the values for ${\mathrm{CSF}}_{\mathrm{ingestion}}$, ${\mathrm{CSF}}_{\mathrm{dermal}}$, and ${\mathrm{CSF}}_{\mathrm{inhalation}}$ were 7.3, 25, and 3.85, respectively [31]. The conversion coefficient for the PAH concentrations is 10^6^. The remaining parameters are described in Table 2.

## 3. Results and Discussion

### 3.1. PAH Concentrations in Farmland Soil

The concentrations of 16 PAHs in the 84 soil samples in autumn and spring are shown in Table 3. The 16 U.S. EPA priority PAHs were detected in all samples. The concentrations of Σ16PAHs in farmland soils in autumn ranged from 27.529 μg/kg to 627.856 μg/kg, with a mean value of 157.531 μg/kg. The mean concentration of the seven carcinogens in autumn was 75.648 μg/kg and ranged from 13.122 μg/kg to 308.086 μg/kg, while Σ7PAHs accounted for 48.02% of the total concentration. The 16 total PAH concentrations in farmland soil in spring varied from 63.826 μg/kg to 1059.767 μg/kg, with a mean value of 258.604 μg/kg, and Σ7PAHs contributed to 39.62%. The results showed that there were seasonal differences in the PAH concentrations in farmland soils, and the PAH concentrations were higher in spring than in autumn, which was related to the climate and precipitation in the study area. During the spring sampling period, there were 2 days with continuous rainfall, which resulted in higher PAH concentrations in the samples that were due to surface runoff and wet deposition. In addition, PAHs in soil are also transferred to plants in various ways. After the autumn harvest, the concentration of PAHs in the soil decreased [33]. Besides that, the photolysis reaction of PAHs may be another influencing factor. Although the spring and autumn temperatures were similar, autumn was less rainy and the sunlight exposure time was longer, resulting in lower soil PAH concentrations in autumn. Some studies have found photolysis of PAHs in surface water, drinking water, and microplastics [34,35,36]. In addition, other scholars have confirmed the photocatalytic degradation of PAHs in the topsoil [37,38,39]. Σ7PAHs with high carcinogenicity account for a higher proportion, which indicates that the safety of farmland soils was more affected.

The concentrations of Σ16PAHs in this study were much lower than those in roadside soils in Delhi, India (the concentration ranged from 1062 μg/kg to 965 μg/kg) and Dhanbad, India (the concentration of 13 PAHs varied from 1019 μg/kg to 10,856 μg/kg) [1,2]. The high concentrations in the study area of India are mainly due to the following factors: (1) there were large traffic loads; (2) traffic congestion was present; (3) low-speed and variable-speed driving conditions occurred; and (4) the surrounding tall buildings affected the diffusion of PAHs. In addition, the PAH concentrations in this study were also lower than for the soils in the urban–rural integration area in Hebei (the concentration ranged from 25 μg/kg to 15,155 μg/kg) and the industrial soils in the Yangtze River Delta (the concentration varied from 189.500 μg/kg to 1070.400 μg/kg) [40,41]. This also suggests that urbanization and industrialization are the main drivers of soil PAH pollution. In addition, traffic flows, road conditions, and the surrounding environments can also affect soil PAH concentrations. The concentrations of Σ16PAHs in this study are similar to those for the rice–wheat continuous cropping soils that are located close to industrial parks in Suzhou, with a value ranging from 125.990 μg/kg to 796.650 μg/kg [42]. Compared with other areas, the farmland soils of Guangzhou were weakly contaminated. The main reason is that the farmland soils examined in this study are located in traditional agricultural areas of Guangzhou and are located far from the industrial area.

### 3.2. Compositional Characteristics of PAHs in Farmland Soils

**Figure 2:**
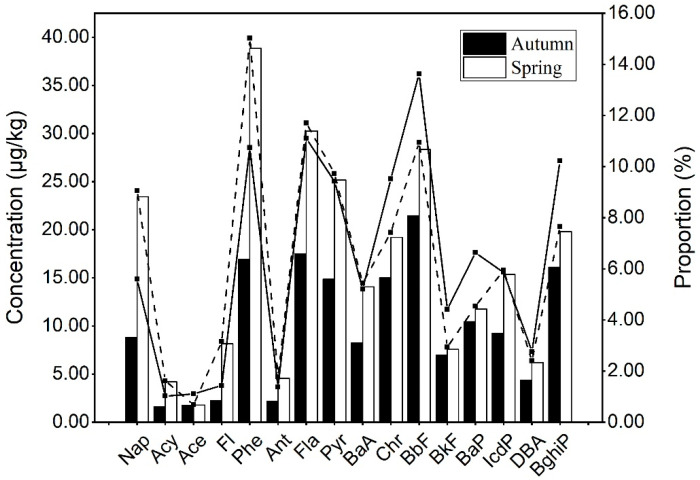
Composition distribution of PAHs in farmland soils in autumn and spring. The bar charts represent the concentration of each PAH. Line charts represent the proportion of each PAH, with the solid black line indicating autumn and the dashed black line indicating spring.

The distributions of the concentrations and proportions of 16 PAHs in the soil in autumn and spring are shown in Figure 2. We can see that the concentration of each PAH in spring was significantly higher than that in autumn. The proportions of each PAH exhibited small differences. The component with the largest proportion was Phe, while the component with the lowest proportion was Ace in spring. BbF accounted for the highest proportion, and Acy accounted for the lowest proportion in autumn.

For the low-molecular-weight (LMW) 2- to 3-ring PAHs, the concentrations of Acy, Ace, Fl, and Ant were low and accounted for approximately 1% of total PAHs. However, the Phe concentrations were highest in autumn and spring, with average concentrations of 16.922 μg/kg and 38.831 μg/kg, accounting for 10% and 15% of the average content of total PAHs, respectively. Nap had the second highest concentrations in autumn and spring, with average concentrations of 8.799 μg/kg and 23.423 μg/kg, comprising 5.60% and 9%, respectively.

For the medium-molecular-weight (MMW) 4-ring and high-molecular-weight (HMW) 5- to 6-ring PAHs, the DBA concentrations in spring were the lowest, with a mean concentration of 6.199 μg/kg, accounting for 2.4% of total PAHs, while the Fla concentration was the highest, with a mean concentration of 30.243 μg/kg and comprised 11.7% of the total PAHs. However, the BbF concentrations in autumn were the highest, with a mean concentration of 21.461 μg/kg, accounting for 13.62% of the total PAHs, while DBA presented the lowest mean concentration, with a concentration of 4.353 μg/kg, which accounted for 2.76% of the total PAHs. Fla, Pyr, Chr, and BghiP also had relatively high concentrations, with values of 17.464 μg/kg, 14.838 μg/kg, 14.993 μg/kg, and 16.097 μg/kg, with proportions of 11.09%, 9.42%, 9.52%, and 10.22%, respectively. Except for Nap and Phe, the concentrations of the LMW PAHs were lower than those of the HMW PAHs.

**Figure 3:**
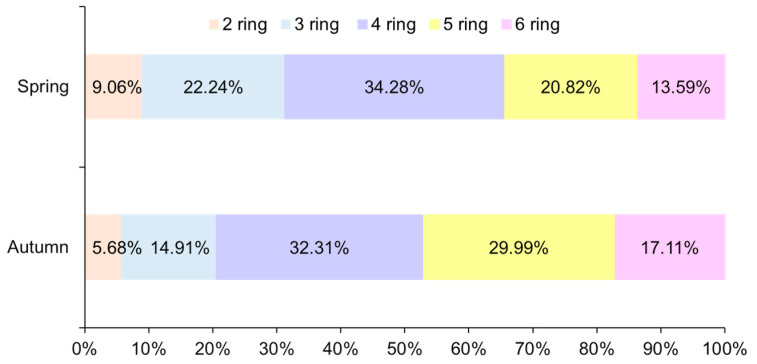
Ring number distribution of the PAHs in farmland soils in autumn and spring.

Regarding the composition of PAHs in farmland soils, there were certain differences in spring and autumn (Figure 3). The 4-ring component was the largest component, which accounted for 32–35%, while the 2-ring component accounted for the lowest amount, which was 5–10%. The PAHs in autumn were dominated by 4-rings, followed by 5-ring and 6-ring, and finally 3-ring. The PAHs in spring were dominated by 4-ring, followed by 3-ring, then 5-ring, and finally 6-rings. In both autumn and spring, the proportion of 4–6-ring PAHs was far larger than that of 2–3-ring PAHs. The overall results were similar to other research results, which show that the ring number composition of PAHs in soil close to the highway was mainly 4–6-ring [2,43]. Medium- and high-molecular-weight PAHs are usually related to the incomplete combustion of petroleum fuels, so the compositions near highways were relatively high. The proportion of 2-ring PAHs in the soil was low, which was related to the molecular weight. The 2-ring PAHs have low molecular weights and easy volatilize into the atmosphere. In addition, in spring, the proportion of 3-ring PAHs was greater than that of 5-ring and 6-ring PAHs, which was related to rainfall. There was more rainfall in spring, and pollutants in the atmosphere entered the soil through runoff and wet deposition, which resulted in higher levels of low-ring components.

### 3.3. Spatial Distribution of PAHs in Farmland Soil

**Figure 4:**
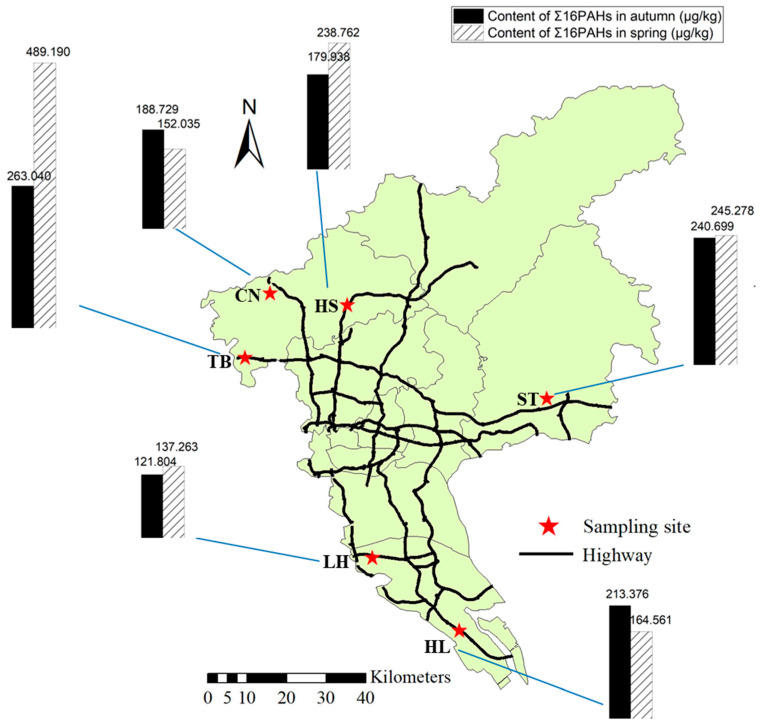
Spatial distribution of PAHs in farmland soil in autumn and spring.

The spatial distributions of the PAHs in farmland soils in autumn and spring are shown in Figure 4. The Σ16PAH contents were relatively high in the TB, ST, and HS areas. Among them, the TB area had the largest Σ16PAH content in spring and autumn, and the Σ16PAH contents were 489.190 μg/kg in spring and 263.040 μg/kg in autumn. In areas ST, HS, HL, and CN, the contents of Σ16PAHs were 245.278 μg/kg, 238.762 μg/kg, 164.561 μg/kg, and 152.035 μg/kg in spring and 240.699 μg/kg, 179.938 μg/kg, 213.376 μg/kg, and 188.729 μg/kg in autumn, respectively. The contents of Σ16PAHs were lowest in the LH area in the two seasons, and the contents were 137.263 μg/kg in spring and 121.804 μg/kg in autumn. Based on the average contents in the two seasons, the maximum content of Σ16PAHs in spring was 489.190 μg/kg, with a minimum content of 137.263 μg/kg. However, the maximum value in autumn was 263.040 μg/kg, and the minimum value was 121.804 μg/kg.

There was a correlation between the concentrations of Σ16PAHs in the farmland soils and traffic loads and road opening times. The opening time of each road is shown in Table 1. We can see that the TB area had an early road opening time and large traffic load, so its PAH concentration was highest. Although the ST area has the latest opening time, the average PAH content was still high. The potential reason was that ST areas were near a national expressway with a large traffic load and sparse roadside trees around the sampling site. Then, the roads in the HS area are open for a long time, the road conditions are complicated, and motor vehicles are always in a state of constant acceleration and deceleration, resulting in higher concentrations. LH has always been a traditional agricultural area in Guangzhou, with lower pollution accumulations, so the concentrations were low.

### 3.4. PAH Concentration in Farmland Soil in Gradient Sampling Area (TB, HL)

**Figure 5:**
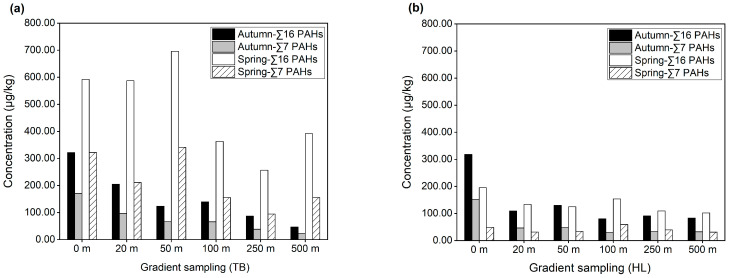
Concentration of PAHs with distance in farmland soil in autumn and spring. (**a**) Gradient sampling area Tanbu Town, Huadu District (TB); (**b**) gradient sampling area Hengli Town, Nansha District (HL).

The highways selected for the gradient sampling area (TB and HL) in this study are Express Highway Round City in Guangzhou and Nansha Port Expressway, respectively. TB is located in the West Second Ring Road of Huadu District, and HL is located in Hengli Town, Nansha District. We set gradients at 0 m, 20 m, 50 m, 100 m, and 250 m from the highway and took the background value at 500 m. The concentrations of PAHs in farmland soil in autumn and spring at different distances are shown in Figure 5.

For the sampling area TB, the Σ16PAH concentration in the farmland soil was higher as a whole, and it was much higher in spring than in autumn. In spring, the overall change trend of Σ16PAH concentration was to increase first and then decrease, reaching a maximum value of 695.789 μg/kg at 50 m. The Σ16PAH concentrations at 0 m and 20 m were similar with little change. From 20 m to 50 m, the concentration of Σ16PAHs increased with distance, while from 50 m to 250 m, the Σ16PAH concentration gradually decreased with the increase in distance, and reached a minimum value of 256.524 μg/kg at 250 m. Meanwhile, the Σ16PAH concentration values at 100 m and 250 m were close to but lower than the background value (391.945 μg/kg) at 500 m. The 16 total PAH concentrations in farmland soil varied from 256.524 μg/kg to 695.789 μg/kg, with a mean value of 489.190 μg/kg, and Σ7PAHs contributed to 45.10%. In autumn, the vertical distances of Σ16PAH concentration from high to low are: 0 m, 20 m, 100 m, 50 m, and 250 m, respectively. The maximum at 0 m was 321.431 μg/kg, and the minimum at 250 m was 86.728 μg/kg. The Σ16PAH concentration decreased gradually with the increase in distance, but increased slightly from 50 m to 100 m. The concentration values at all distances were higher than the background value (46.403 μg/kg) at 500 m. The average content of 16 PAHs was 263.040 μg/kg, and Σ7PAHs accounted for 50.14% of the total.

From the results, we can see that highway traffic emissions have a great impact on the concentration of PAHs in farmland soil. In terms of seasons, the pollution in spring is much higher than that in autumn, and the concentration of PAHs in spring is about twice or more than that in autumn. The main reason for this is not only the impact of rainfall, but also closely related to crop planting and farmland soil ploughing [33,44,45,46]. In autumn, the concentration of PAHs gradually decreases with the increase in the distance from the highway. This is because under the action of natural conditions such as wind speed and wind direction, the farther the distance is, the less aerosols there are, and the PAHs deposited into the soil through wet and dry deposition will be reduced. In spring, the concentration of PAHs is the highest at 50 m away from the highway, followed by 0 m and 20 m, and then gradually decreases with the increase in distance. We found that the closer to the highway, the more serious the pollution. This is consistent with previous research conclusions [25,26,47,48]. PAH concentrations at 0 m and 20 m were lower than those at 50 m, which was because the luxuriant branches and leaves of the roadside trees in spring hindered the diffusion of pollutants. The highest concentration at 50 m is mainly due to the transfer of particulate matter and aerosols generated by vehicle exhaust and the impact of runoff after heavy rainfall [49,50,51,52]. An interesting phenomenon is that the background value of PAHs at 500 m in spring is higher than that at 100 m and 250 m, which indicates that the background value of soil PAHs in the sampling area is high and the soil pollution is serious. The main reason is that Express Highway Round City in Guangzhou is connected with many expressways, national highways, and provincial trunk roads, sharing a large amount of transit traffic, and the traffic flow is large and mostly heavy vehicles. Previous studies have confirmed that heavy traffic flow and traffic congestion lead to high PAH emissions [2,47,53]. In addition, another possible reason is that there is a small ditch not far from the sampling site of the background value, and more rainfall in spring will also have a certain impact on it.

Compared with sampling area TB, the PAH concentration in sampling area HL was lower as a whole. This is because the Nansha District where the Nansha Port Expressway is located is mainly arable land and ecological land, and the traffic flow on the road is low. Except for 0 m and 100 m, the concentrations in spring and autumn were similar. In spring, the concentrations of 16 PAHs all decreased with the increase in distance, except for the increasing trend from 50 m to 100 m. The highest concentration at 0 m was 195.584 μg/kg, followed by 100 m, with a concentration of 153.664 μg/kg, and a minimum concentration of 109.563 μg/kg at 250 m, which was close to the background value at 500 m. In autumn, the concentration at 0 m is also the highest, with a value of 317.821 μg/kg, which is much higher than that in spring. The PAH concentrations showed a decreasing trend from 0 to 20 m and 50 to 100 m, while increasing from 20 to 50 m and 100 to 250 m. The Σ16PAH concentration was the smallest at 100 m, with a value of 80.423 μg/kg, which was close to the background value. On the whole, in spring and autumn, except for the high concentration of PAHs at 0 m, other gradient concentrations have little change, which are close to the background value at 500 m. This is mainly because the soil taken at 0 m is close to the expressway toll station, at which traffic congestion and frequent vehicle speed change result in high PAH concentration. In addition, PAHs emitted from tire debris and asphalt pavement also accumulate in roadside soil [50,54]. At the same time, after rainfall, the water on the highway spills into the soil, which will also affect the PAH concentration in the adjacent soil [49].

### 3.5. PAH Source Identification

**Figure 6:**
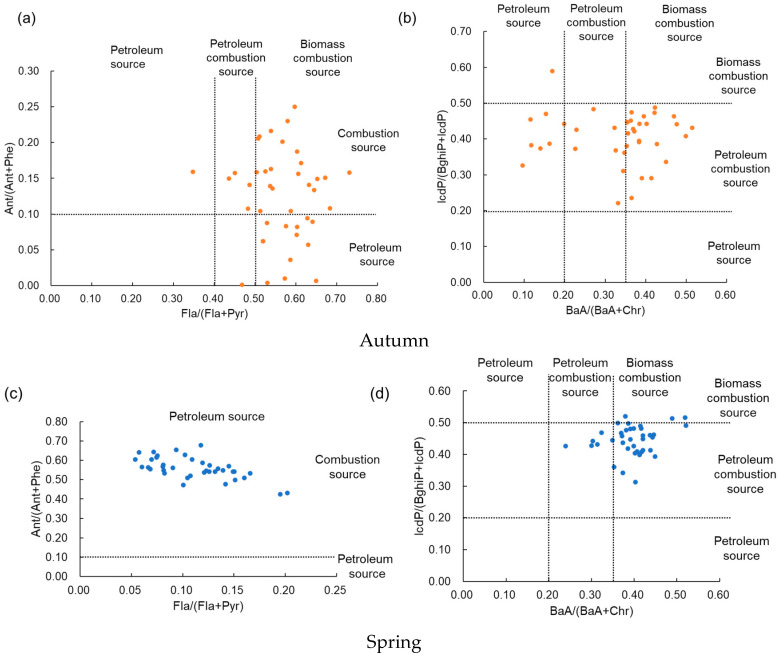
Diagnostic ratios of PAHs in the farmland near the highway in different seasons. (**a**,**b**) Autumn and (**c**,**d**) spring.

The ratios of BaA/(BaA + Chr), Fla/(Fla + Pyr), Ant/(Ant + Phe), and IcdP/(BghiP + IcdP) were used to characterize the potential sources of PAHs (Figure 6). Except for one sampling point, the values of Fla/(Fla + Pyr) at the other points were greater than 0.4 in autumn, indicating that the main source of PAHs consisted of combustion sources. However, the values at all points in spring were less than 0.4, which suggested that the main source of PAHs was petroleum [55]. In autumn, the Ant/(Ant + Phe) ratios in 32.5% of sampling points were less than 0.1, which indicated that the PAHs originated from petroleum sources. However, in 67.5% of sampling points in autumn and at all points in spring, the Ant/(Ant + Phe) ratios are greater than 0.1, which means that the source of PAHs was petroleum combustion [56]. The IcdP/(BghiP + IcdP) ratios for almost all sampling points in autumn and 92% of sampling points in spring are greater than 0.2 and less than 0.5, indicating that the main source of PAHs consisted of petroleum combustion [57]. In addition, based on the BaA/(BaA + Chr) ratios, we found that 60% of sampling points in autumn and 84% in spring had values above 0.35, which indicated that the main source was biomass burning. At 20% of sampling points in autumn and 18% in spring, the BaA/(BaA + Chr) ratios were between 0.2 and 0.35, indicating that PAHs were mainly derived from petroleum combustion sources [58,59].

Overall, the PAHs in farmland soil mostly come from mixed sources of petroleum and biomass combustion.

### 3.6. Pollution Level Assessment of Soil PAHs

The methods proposed by Maliszewska-Kordybach (1996) were used to more rigorously assess farmland soil pollution [60]. There were four levels of soil pollution by PAHs: ΣPAH concentrations less than 200 μg/kg indicated uncontaminated; those between 200 and 600 μg/kg indicated weakly contaminated; ΣPAH concentrations from 600 to 1000 μg/kg indicated contaminated; and values greater than 1000 μg/kg suggested heavily contaminated. Based on these criteria, 40.48% of the samples were considered to be weakly contaminated and 59.52% could be considered as uncontaminated (Table 4).

We also used the Nemerow index method to assess the pollution levels of PAHs in soils based on the evaluation criteria obtained from the Canadian government. According to the pollution index p, these levels are divided into five evaluation grades. A value of *p* less than or equal to 0.7 is safe. A value of *p* from 0.7 to 1 suggests a warning line, a value between 1 and 2 indicates weak contamination, a value from 2 to 3 indicates contamination, and a value greater than 3 indicates heavy contamination. Our results showed that the *p* values of all soil samples were below the security level (Table 5).

### 3.7. Health Risk Assessment

#### 3.7.1. Toxic Equivalence Concentration

Different individual PAHs have different toxic effects, so the toxic equivalence factors (TEFs) were used to determine the toxic equivalence concentration (TEQ_BaP_) of soil PAHs [61]. Table 6 shows the TEF values of PAHs and the TEQ_BaP_ concentrations. The total TEQ_BaP_ of 16 PAHs in farmland soil samples varied from 5.068 μg/kg to 79.380 μg/kg, with an average value of 22.420 μg/kg. The total TEQ_BaP_ values for the seven carcinogenic PAHs were between 4.910 μg/kg and 77.828 μg/kg, with a mean value of 21.894 μg/kg, and contributed 98.62% for the total TEQ_BaP_. The results suggested that the total carcinogenicity of soil PAHs was mainly caused by seven carcinogenic PAHs; among them, BaP contributed the most, followed by DBA, which accounted for 49.58% and 23.46% of the total TEQ_BaP_ of soil PAHs, respectively. The 16 PAHs in farmland soil samples had TEQ_BaP_ values that were lower than the soil pollution risk management and control for agricultural land standard value of 0.55 mg/kg. Therefore, the soil in the study area poses little risk to human health.

#### 3.7.2. Exposure Model

Ingestion, dermal contact, and inhalation are three ways that the human body comes into contact with soil pollutants. Due to the physical differences at different ages, we calculated the lifetime cancer risks of adults and children separately in this study. The results shown in Table 7 were obtained through Equations (1)–(3). When combining the three exposure methods, in all soil samples, the range of ILCRs for children was between 9.68 × 10^−5^ and 1.09 × 10^−3^, with a mean of 3.44 × 10^−4^, while the range for adults was 5.77 × 10^−5^ to 6.48 × 10^−4^, with a mean of 2.05 × 10^−4^. The total ILCRs of both children and adults exceeded 10^−6^, which indicated a low risk or critical health level in the soil. Children and adults bear different risks for different exposure pathways. The risk caused by inhalation was the lowest, but the risk from dermal contact was the highest. Other scholars have also found that the dermal contact exposure route of PAHs is higher risk than the inhalation and ingestion routes [62]. Overall, under the current concentrations, the soil PAH exposure risk in children was greater than that in adults, and the mean of the total ILCRs was approximately 1.68 times that in adults. The ILCRs were higher for children than adults because of their different individual differences and lifestyle habits [29,63]. Studies have found that children are more likely to ingest contaminated soil because of their hand to mouth activities [13,64]. In addition, children also have higher PAH intake than adults due to their lower body weight, so they are at greater risk [41].

## 4. Conclusions

Vehicle exhaust is an important source of roadside soil PAHs. In this study, 16 PAHs in the farmland soils of Guangzhou that were located near different highways in different seasons were investigated. The farmland soils in rapidly urbanized areas are seriously polluted by PAHs. Different highway conditions have different degrees of influence on soil PAH pollution. We found that factors such as traffic loads, traffic congestion, driving conditions, road opening times, and road surroundings significantly affected the concentrations and distributions of PAHs in farmland soils. When the traffic load is heavy, the opening time is early, the roadside trees around the road are sparse, and the driving conditions are often low speed or variable speed, the PAH concentrations in farmland soils were higher, such as in the sampling TB, ST, and HS areas. In the traditional agricultural areas of Guangzhou, there was lower pollution accumulation, so the PAH concentrations in the LH sampling area were the lowest. Besides that, rainfall conditions, farmland tillage, photolysis reaction, and surface runoff also affected the of PAH concentrations in farmland soils, which resulted in seasonal differences in soil PAH concentrations. The soil PAH concentrations in the study area were much higher in spring than in autumn. In addition, the distance from the highway was also closely related to the concentration of soil PAHs. Gradient sampling studies found that the concentration of soil PAHs decreased gradually with the increase in distance. The closer to the highway, the more serious the pollution. Finally, this research determined that the overall potential ecological risk of the farmland soils in Guangzhou is low, but continuous monitoring is still required to assess the impact of transportation emissions on the environment and human health.

## Figures and Tables

**Table 1:** Specific information for the six sampling areas.

Sample Serial Number	Location	Date	Number of Samples	Nearby Highway	Road Opening Time
CN	Chini Town, Huadu District, Guangzhou	October 2020 (Autumn) March 2021 (Spring)	10	Pearl River Delta Ring Expressway	December 2014
TB	Tanbu Town, Huadu District, Guangzhou		22	Express Highway Round City in Guangzhou	December 2006
HS	Huashan Town, Huadu District, Guangzhou		10	Da Guang Expressway	January 2002
ST	Shitan Town, Zengcheng District, Guangzhou		10	Jinan Guangzhou Expressway	December 2015
HL	Hengli Town, Nansha District, Guangzhou		22	Nansha Port Expressway	December 2004
LH	Lanhe Town, Nansha District, Guangzhou		10	Express Highway Round City in Guangzhou	December 2010

**Table 2:** Parameters used for lifetime carcinogenic risk assessment (ILCR).

Parameter	Units	Child	Adult	References
Body weight (BW)	kg	6.94	58.55	[32]
Exposure frequency (EF)	day year^−1^	350	350	[27]
Exposure duration (ED)	year	6	24	-
Inhalation rate $\left({\mathrm{IR}}_{\mathrm{inhalation}}\right)$	m^3^ day^−1^	5.65	13.04	[32]
Dust ingestion rate $\left({\mathrm{IR}}_{\mathrm{ingestion}}\right)$	mg day^−1^	200	100	[27]
Dermal exposure area (SA)	cm^2^ day^−1^	2800	5700	[27]
Dermal exposure factor (AF)	mg cm^−2^	0.20	0.07	[27]
Dermal adsorption factor (ABS)	-	0.13	0.13	[30]
Average lifespan (AT)	day	81.34 × 365	81.34 × 365	GDASS, 2018
Particle emission factor (PEF)	m^3^ kg^−1^	1.36 × 10^9^	1.36 × 10^9^	[30]

Notes: GDASS refers to the Guangdong Academy of Social Sciences.

**Table 3:** The concentrations of 16 PAHs in farmland soils in autumn and spring.

PAHs (μg/kg)	Rings	Autumn			Spring		
		Max	Min	Mean	Max	Min	Mean
Nap	2	28.954	3.897	8.799	78.143	5.667	23.423
Acy	3	10.582	0.000	1.613	14.111	0.686	4.202
Ace	3	6.040	0.000	1.745	4.429	0.504	1.768
Fl	3	6.653	0.357	2.249	33.545	2.049	8.148
Phe	3	58.210	3.527	16.922	159.557	9.849	38.831
Ant	3	5.900	0.049	2.155	12.635	1.185	4.569
Fla	4	55.711	3.709	17.464	112.717	8.021	30.243
Pyr	4	49.709	2.509	14.838	98.515	8.807	25.153
BaA	4	27.773	0.674	8.214	103.496	2.742	14.068
Chr	4	51.425	3.474	14.993	96.026	5.237	19.196
BbF	5	77.455	5.749	21.461	96.006	7.876	28.326
BkF	5	27.223	1.102	6.952	37.626	2.195	7.568
BaP	5	54.226	1.609	10.450	72.046	1.239	11.753
DBA	5	25.860	0.000	4.353	22.614	0.591	6.199
IcdP	6	44.124	0.515	9.226	50.395	3.224	15.364
BghiP	6	98.010	0.358	16.097	67.904	3.953	19.792
Σ16PAHs	-	627.856	27.529	157.531	1059.767	63.826	258.604
Σ7PAHs	-	308.086	13.122	75.648	478.210	23.105	102.475

Notes: Σ16PAHs refer to the total concentration of 16 PAHs. Σ7PAHs represents the concentration of 7 carcinogenic PAHs (e.g., BaA, Chr, BbF, BkF, BaP, IcdP, and DBA).

**Table 4:** Evaluation standard for the total pollution levels of PAHs.

Level	Total Concentration Range of PAHs	Number of Samples
Uncontaminated	<200 μg/kg	25
Weak contamination	200~600 μg/kg	17
Contamination	600~1000 μg/kg	0
Heavy contamination	>1000 μg/kg	0

**Table 5:** Statistics of the Nemerow index of soil PAHs.

Level	Safe (*p* ≤ 0.7)	Warning Line (0.7 < *p* ≤ 1)	Weak Contamination (1 < *p* ≤ 2)	Contamination (2 < *p* ≤ 3)	Heavy Contamination (*p* > 3)
Number of samples	42	0	0	0	0

**Table 6:** Toxic equivalence concentration (TEQ_BaP_) of soil PAHs.

PAHs (μg/kg)	TEFs	Min	Max	Mean
Nap	0.001	0.007	0.044	0.016
Acy	0.001	0.001	0.007	0.003
Ace	0.001	0.000	0.005	0.002
Fl	0.001	0.001	0.017	0.005
Phe	0.001	0.091	0.831	0.277
Ant	0.010	0.001	0.007	0.003
Fla	0.001	0.010	0.065	0.024
Pyr	0.001	0.008	0.057	0.020
BaA	0.100	0.311	5.441	1.099
Chr	0.010	0.057	0.542	0.170
BbF	0.100	0.853	6.415	2.473
BkF	0.100	0.187	2.285	0.722
BaP	1.000	2.137	40.936	11.006
IcdP	0.100	0.283	3.566	1.215
DBA	1.000	1.082	18.644	5.208
BghiP	0.010	0.039	0.521	0.177
16 PAHs	-	5.068	79.380	22.420
7 PAHs	-	4.910	77.828	21.894

**Table 7:** The ranges of lifetime carcinogenic risk assessment (ILCR) in children and adults with three exposure pathways.

Exposure Pathway	Children			Adults		
	Min	Max	Mean	Min	Max	Mean
Dermal contact	5.370 × 10^−5^	6.030 × 10^−4^	1.906 × 10^−4^	3.693 × 10^−5^	4.147 × 10^−4^	1.311 × 10^−4^
Ingestion	4.308 × 10^−5^	4.837 × 10^−4^	1.529 × 10^−4^	2.079 × 10^−5^	2.335 × 10^−4^	7.379 × 10^−5^
Inhalation	4.720 × 10^−10^	5.299 × 10^−9^	1.675 × 10^−9^	1.051 × 10^−9^	1.181 × 10^−8^	3.731 × 10^−9^
Total	9.678 × 10^−5^	1.087 × 10^−3^	3.435 × 10^−4^	5.772 × 10^−5^	6.482 × 10^−4^	2.049 × 10^−4^
